# Strengthening of Back Muscles Using a Module of Flexible Strain Sensors

**DOI:** 10.3390/s150203975

**Published:** 2015-02-09

**Authors:** Wan-Chun Chuang, Hwai-Ting Lin, Wei-Long Chen

**Affiliations:** 1 Department of Mechanical and Electromechanical Engineering, National Sun Yat-sen University, 70 Lienhai Rd., Kaohsiung 80424, Taiwan; E-Mail: f24930329@gmail.com; 2 Department of Sports Medicine, Kaohsiung Medical University, Kaohsiung 80708, Taiwan; E-Mail: whiting@kmu.edu.tw

**Keywords:** flexible sensor, back muscles, muscle strengthening

## Abstract

This research aims at developing a flexible strain module applied to the strengthening of back muscles. Silver films were sputtered onto flexible substrates to produce a flexible sensor. Assuming that back muscle elongation is positively correlated with the variations in skin surface length, real-time resistance changes exhibited by the sensor during simulated training sessions were measured. The results were used to identify the relationship between resistance change of sensors and skin surface stretch. In addition, muscle length changes from ultrasound images were used to determine the feasibility of a proof of concept sensor. Furthermore, this module is capable of detecting large muscle contractions, some of which may be undesirable for the prescribed training strategy. Therefore, the developed module can facilitate real-time assessments of the movement accuracy of users during training, and the results are instantly displayed on a screen. People using the developed training system can immediately adjust their posture to the appropriate position. Thus, the training mechanism can be constructed to help user improve the efficiency of back muscle strengthening.

## 1. Introduction

According to statistics, people spend an average of 9.3 h sitting per day, which induces some serious health consequences, such as backache, high cholesterol, diabetes, and obesity. Backache often occurs during one's lifetime, while a sedentary lifestyle can aggravate the injury. In most cases of back pain, one can benefit from exercises to strengthen one's back muscles. Therefore, exercising the back muscles in daily life is an effective way to prevent backache.

In 2003, Engel [1] proposed a technique to produce flexible sensors, and studied the strain *vs.* changes in resistance. In 2004 and 2009, Shimojo [2] and Peng [3], respectively, proposed a capacitive flexible sensor and analyzed the relationship between changes in pressure and resistance. In 2007, Yang [4] presented a flexible and implantable device to provide high-resolution mechanical strain data from a bone surface. This Parylene-based strain gauge combined with a wireless telemetry circuit can be used to monitor the real-time surface deformation of a bone. In 2014, Ghosh [5] reported the development of a flexible ammonia sensor. The sensing element is based on chemically derived multilayered graphene deposited on a 3-inch diameter filter paper. Babu [6] produced a piezoelectric flexible sensor with enhanced electromechanical properties. This sensor, which incorporated carbon nanotubes (CNTs) and carbon black (CB) was fabricated by a solution casting technique using a constant PZT/PDMS ratio of 40/60 and conductive fillers ranging from 0 to 0.5 vol.%. Weltin [7] developed a versatile microsensor platform for *in vivo* applications monitoring neurotransmitters and energy metabolism. In contrast to systems made from silicon or ceramic, the fully flexible sensor strip is based on a polymer substrate and can be fabricated in multiple geometries. In 2015, Nabar [8] produced flexible, bendable tactile pressure sensors with crystalline ZnO nanorods as a robotic skin or as part of a multimodal sensing garment. The sensors are arranged in addressable arrays for detection of spatial tactile pressure variation with a resolution of 1 mm or better. Previous literature has shown different kinds of fabrication processes to produce flexible sensors, and analyzed their physical properties, but flexible sensors had not been used for muscle strengthening before.

Several systems had been developed for muscle strengthening training. Gruner [9] developed a system for evaluation and exercise-conditioning of paralyzed leg muscles in 1983. A computer-controlled electrical stimulation system, using surface electrodes, automatically regulated the sets of leg extension exercises. Load weights attached just above the ankles can be progressively increased over a number of training sessions in such a manner that a measure of the fitness of the legs can be obtained. In 2006, Yang [10] developed an enhanced rehabilitation and assessment system for people with impaired leg muscles. The system consists of four major parts: sensory and signal conversion circuits convert the lever arm lengths and muscle strengths of the leg into a proper electronic signal and then deliver the signal to the computer. Then, the interactive interface design lets a trainee complete the training process independently without the involvement of medical staff. In 2007, Miyoshi [11] presented a rehabilitation system utilising a haptic device. This system aimed to integrate motion and sensory therapy ensuring that the patient's interest is maintained and to establish a quantitative assessment of the level of disorder. This system consisted of a haptic device, a computer and a LCD monitor. The user moved the grip of the haptic device according to the training program, which was displayed on the computer screen.

Various scholars have applied ultrasound images in muscle exercise. Shear wave elastography is an increasingly popular ultrasound technique for evaluating the mechanical properties of skeletal muscle tissue. In 2014, Akagi [12] investigated the muscle hardness of the triceps brachii (TB) before and immediately after a resistance exercise. Results showed that muscle hardness was significantly higher at 70% of the upper arm length than at the other regions before and after a resistance exercise. Hoffrén-Mikkola [13] examined the effects of repetitive hopping training on muscle activation profiles and fascicle–tendon interaction in the elderly. Alegre [14] analyzed the muscle adaptations induced by two protocols of isometric training performed at different muscle lengths. It was mentioned that isometric training at specific knee angles led to significant shifts of peak torque in the direction of the training muscle lengths. Konrad [15] investigated the influence of a six-week static stretching training program on the structural and functional parameters of the human gastrocnemius medialis muscle and the Achilles tendon. In 2015, Eby [16] quantified the passive stiffness, or shear modulus, of the biceps brachii throughout adulthood in flexed and extended elbow positions, and established normative trends for skeletal muscle shear modulus throughout adulthood.

In 1993, Dolan [17] found the spine anteflexion angles and erector spinae strengthening were related. As spine anteflexion angles increase, the epidermis also extends. Thus, we assumed that the extension of the epidermis has a positive correlation with muscle contraction to determine muscle strengthening levels. Therefore, the purpose of this study was to develop a flexible strain sensor module that can detect the skin stretch that could be applied for back muscle strengthening. Moreover, the relationship between changes in strain module resistance and epidermal length was also analyzed during the training process. The data was used to establish an effective training system, and the system was expected to generate appropriate strengthening of back muscles.

## 2. Research Methods

The research methods comprised four steps: (1) the fabrication process of the flexible strain sensor; (2) skin stretch measurement during back muscle training; (3) the resistance variation of sensor during roman chair training; (4) determination of training system feasibility.

### 2.1. Fabrication Process of Flexible Strain Sensor

**Figure 1.**
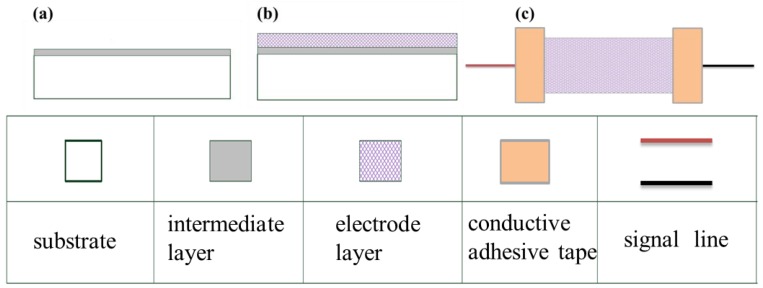
Fabrication process of the flexible strain sensor.

For the flexible sensor fabricated in this study, artificial skin (PAUL HARTMANN Limited, Heywood, UK) that consists mainly of hydrophilic colloids and adhesive polymers was used as the substrate. The flexible sensor was fabricated by sputtering a thin film of silver on the substrate. The parameter settings of the radio frequency (RF) sputtering machine are shown in Tables 1 and 2. Figure 1 shows the fabrication process of flexible strain sensor. Chromium was first coated as an interlayer on the substrate to increase the adhesion between the electrode layer and the substrate (Figure 1a). Subsequently, silver was coated as the electrode layer (Figure 1b). Finally, the signal line is connected with the electrode layer by conductive adhesive tape. The statistical size and dimensions of the sensor are shown in Table 3.

### 2.2. Skin Stretch Measurement during Back Muscle Training

**Figure 2.**
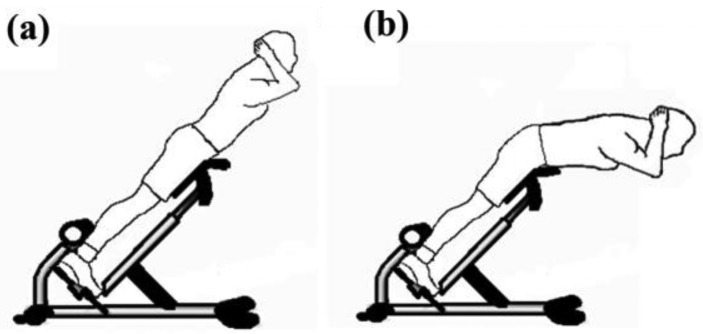
Roman chair training process: (**a**) preparation posture; (**b**) flexion.

**Figure 3.**
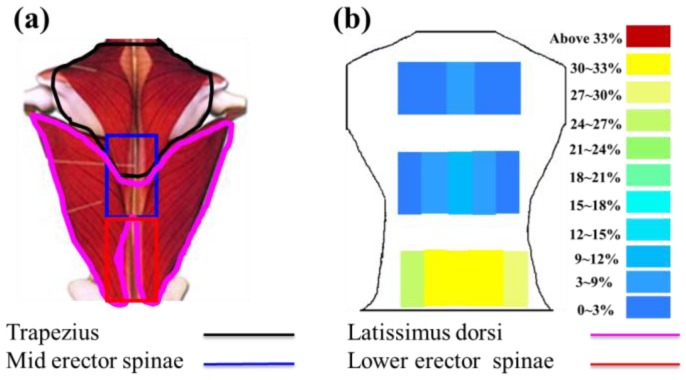
(**a**) Names of the muscles that correspond to the sensory positions; (**b**) the skin stretch at each test position.

**Figure 4.**
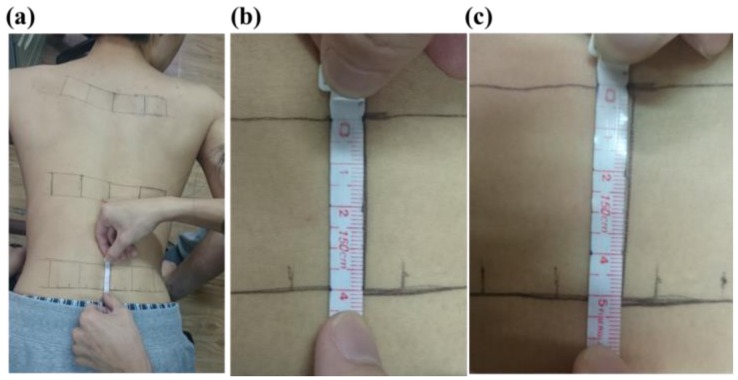
Marked line for each test position during the Roman chair training process: (**a**) measured position; (**b**) preparation posture; (**c**) flexion.

To measure the skin stretch during back muscle strengthening, the back extension exercise on the Roman chair was used as an example to test (Figure 2). During the Roman chair training process, the upper body must be kept straight. This training process uses the trapezius, latissimus dorsi, and the erector spinae. Consequently, this study focused on measuring the skin stretch of these muscles' related locations. The anthropometric data of this participant are shown in Table 4. The test positions for the trapezius, latissimus dorsi, and erector spinae (mid and lower) epidermal extension strain are shown in Figure 3a. Measuring the change of marked line for each test position during the Roman chair training process, the strain can be obtained (Figure 4). Figure 3b shows the skin stretches in different positions during the Roman chair training process and indicated in different colors.

### 2.3. The Resistance Variation of Sensor during Roman Chair Training

**Figure 5.**
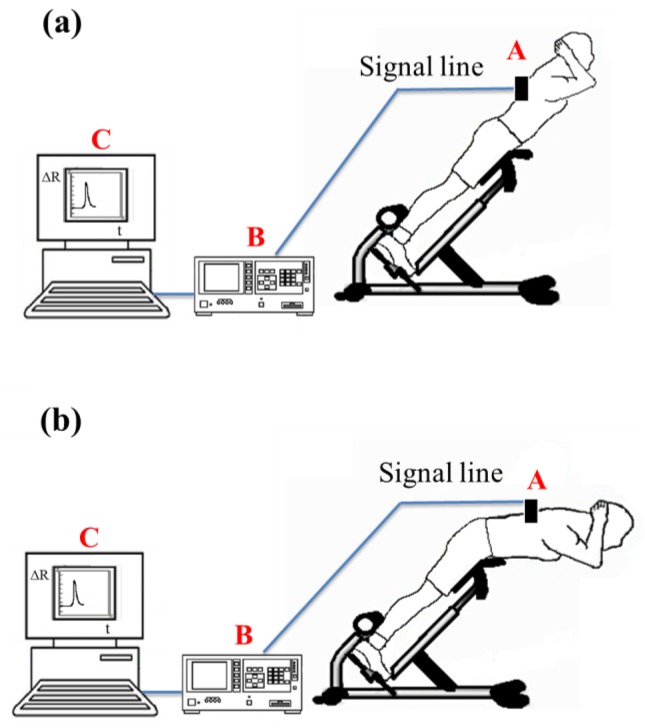
Experimental setup for Roman chair training: (**a**) preparatory action; (**b**) anteflexion. (**A**: flexible strain sensor, **B**: data acquisition unit 34970A, **C**: computer)

**Figure 6.**
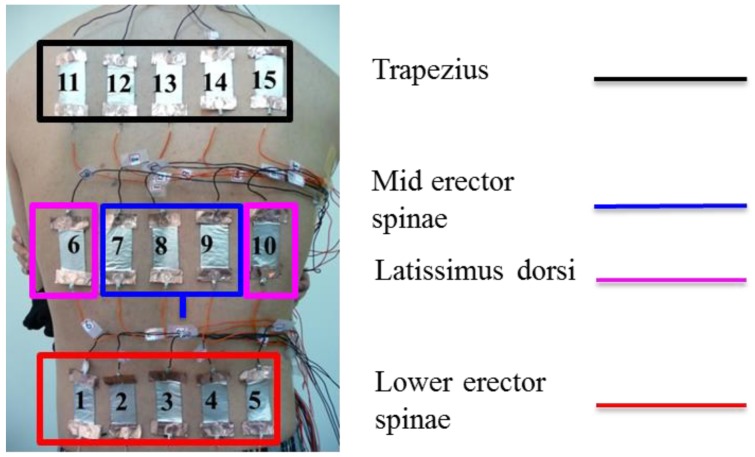
Test positions of flexible strain sensor array.

**Figure 7.**
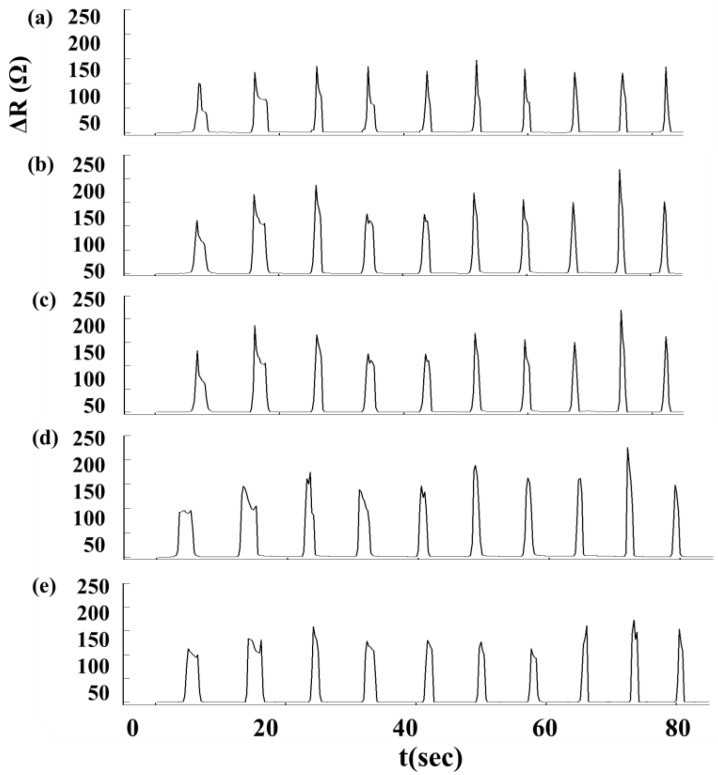
Changes in sensor resistance during 10 repetitions targeting the lower erector spinae: (**a**) position 1; (**b**) position 2; (**c**) position 3; (**d**) position 4; (**e**) position 5.

**Figure 8.**
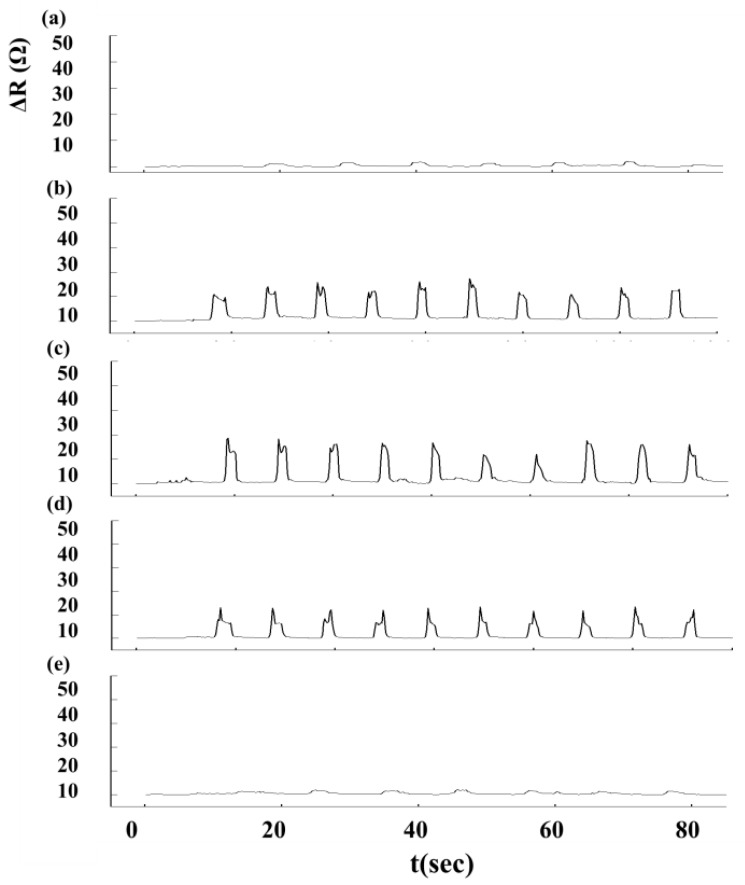
Changes in sensory resistance during 10 repetitions targeting the latissimus dorsi and mid erector spinae: (**a**) position 6; (**b**) position 7; (**c**) position 8; (**d**) position 9; (**e**) position 10.

**Figure 9.**
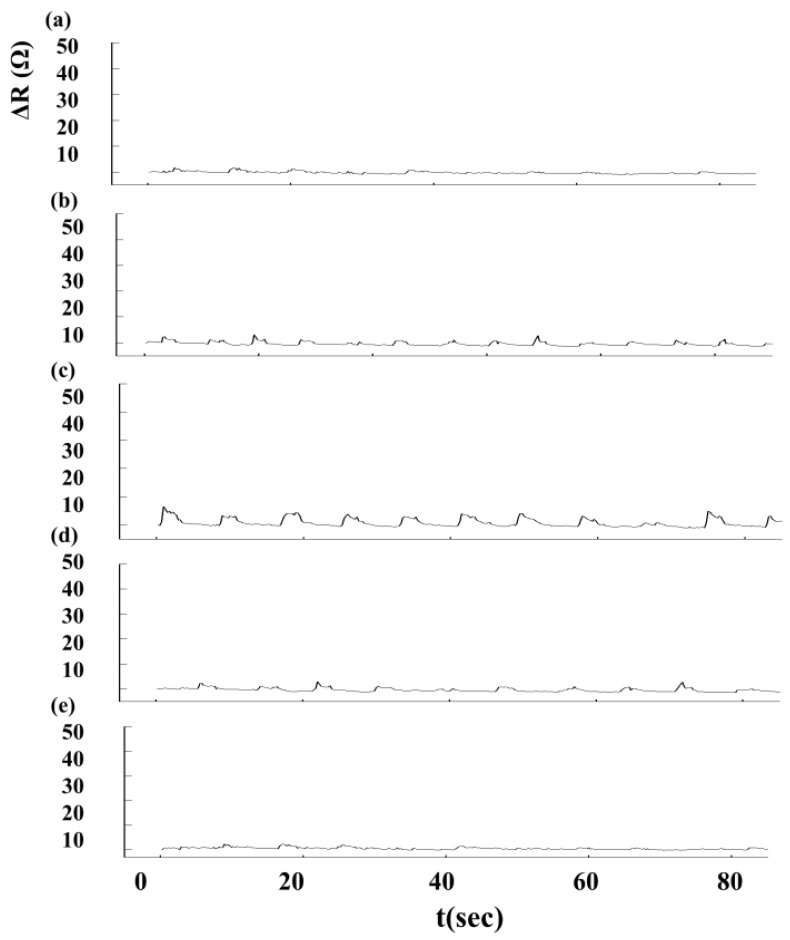
Changes in sensor resistance during 10 repetitions targeting the trapezius: (**a**) position 11; (**b**) position 12; (**c**) position 13; (**d**) position 14; (**e**) position 15.

We used an Agilent 34970A data acquisition unit (Agilent Technologies, Inc, Santa Clara, CA, USA) to measure changes in sensor resistance and recorded the relationship between changes in resistance and time. The experimental setup for Roman chair training is shown in Figure 5. During the Roman chair training process, we used 15 sets of flexible sensors designed for this study, as shown in Figure 6. These sensors were used to measure the change in resistance caused by skin stretch at the test positions. Figures 7, 8 and 9 show the changes in sensor resistance for the lower erector spinae, latissimus dorsi, mid erector spinae, and trapezius during 10 repetitions of hyperextension on Roman chair. Table 5 shows the skin stretch, changes in average resistance, and standard deviation data from Figures 7, 8 and 9.

### 2.4. Determination of Training System Feasibility

**Figure 10.**
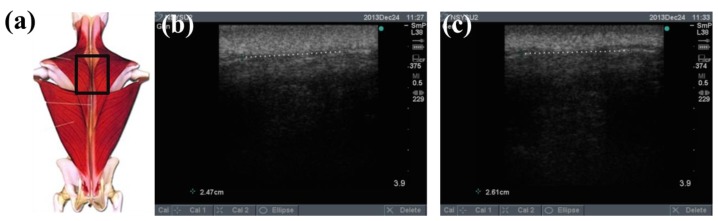
Results from ultrasound images: (**a**) measured trapezius position; (**b**) preparatory action; (**c**) anteflexion action.

**Figure 11.**
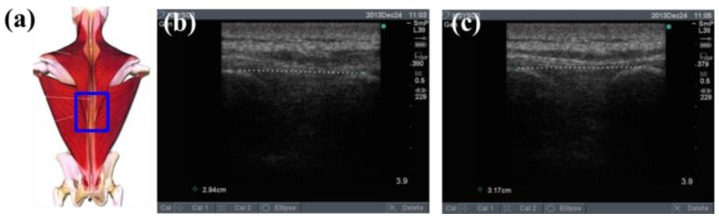
Results from ultrasound images: (**a**) measured mid erector spinae position; (**b**) preparatory action; (**c**) anteflexion action.

**Figure 12.**
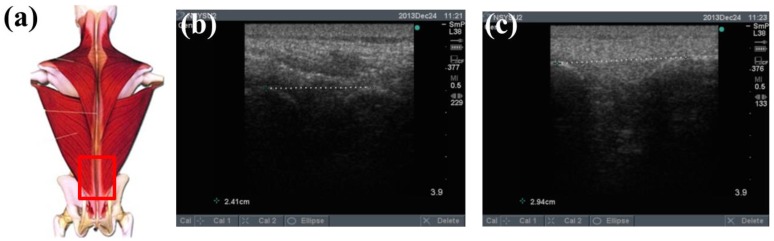
Results from ultrasound images: (**a**) measured lower erector spinae position; (**b**) preparatory action; (**c**) anteflexion action.

To determine the feasibility of the training system proposed in this study, we used ultrasound imaging to observe the muscles' length changes during contraction and determined whether these changes were correlated with skin stretches. Ultrasound image measurement is used for exploring the human body, such as observing tissue structure, blood or tissue movement, and the mechanical characteristics of tissues. Tissue structure is mainly observed using grayscale images. The primary principle in ultrasound imaging is the reflection of ultrasound waves when the waves encounter different density interfaces in the human body. The piezoelectric crystals in the sensor head receives the signals and converts the signals into images according to the direction, distance, and strength of the waves. Ultrasound imaging is often used to examine tumours, tissues, or organs inside the body. Moreover, ultrasound images are also usually used to exam the muscle and soft tissue in rehabilitation and orthopaedic departments [12–16]. The SonoSite Titan ultrasound system (FUJIFILM SonoSite, Inc, Bothell, WA, USA) was used to measure the muscle length changes during exercise. The trapezius, mid erector spinae, and lower erector spinae were measured. The ultrasound image of the muscles at different trunk angles during Roman chair back hyperextension training is shown in Figures 10, 11 and 12, the (b) component showed the image in the preparatory actions and (c) component showed the image in the flexion 45 degrees. The muscle length in the specific positions is shown in Table 6. The lower erector spinae shown in Table 6 had a muscle shortening length of 0.53 cm (the largest length change). The test position correspond to skin stretch of 20%–35%, as shown in Figure 3b, was the largest change among the three positions. The results showed that during Roman chair back hyperextension training, changes in back skeletal muscle extension length exhibited a positive correlation changes in skin stretch. Therefore, the feasibility of a proof of concept sensor has been demonstrated.

## 3. Result and Discussion

### 3.1. The Resistance Variation of Sensor vs. the Skin Stretch

**Figure 13.**
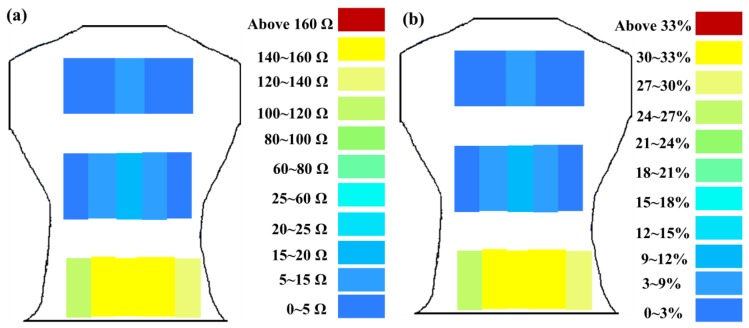
(**a**) Resistance changes measured by flexible sensor arrays; (**b**) skin stretch.

The greater the resistance changes measured by sensors, the greater the changes in skin stretch. The resistance changes measured by flexible sensor arrays are shown in Figure 13. Figure 13a shows the resistance change measured by flexible sensor arrays and Figure 13b shows the skin stretch. The similar changes pattern of Figure 13a,b conform to the concept of Roman chair hyperextension training, which trains the lower erector spinae primarily, this position also yielded the greatest resistance change. The latissimus dorsi and mid erector spinae also have training effect and yielded the second highest resistance change. While the trapezius had least training effect and yielded the smallest. It demonstrates that resistance changes measured by flexible sensor arrays had a positive correlation with skin stretch.

### 3.2. Comparison of Testing Methods for Back Muscle Strengthening Levels

Typically, due to the sensors size of ultrasound images, the device can only measure partial limited ranges. Ultrasound images have to find a significant anatomical marker to quantify the muscle length change. On the contrary, the flexible sensor developed in this study is lower cost than ultrasound images due to the fact that the device has the advantages of being cheap and very easy to produce.

### 3.3. Estimation of Correct Posture for Roman Chair Training

**Figure 14.**
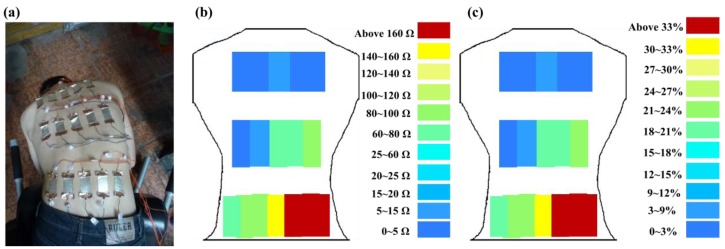
Incorrect posture: (**a**) measured positions; (**b**) resistance changes measured by flexible sensor; (**c**) corresponding skin stretch.

Figure 14b,c shows the resistance changes of flexible sensor arrays and corresponding skin stretch during Roman chair back training while incorrect posture (side bending posture) (Figure 14a). Comparison of Figures 13 and 14 shows that when the training position was incorrect, the resistance changes on the two side spine muscles were clearly asymmetrical. Furthermore, the greatest change primarily occurred in the muscle training range (the lower erector spinae). This position had the highest resistance changes, the latissimus dorsi and mid erector spinae had the second highest resistance changes, and the trapezius had the smallest. It demonstrates that resistance changes detected by flexible sensor arrays can show whether Roman chair back training movements are correct or not.

## 4. Conclusions

In this paper, flexible sensor arrays have been successfully developed for the back muscle training process. A highly positive relationship between changes in resistance and the corresponding skin stretches was found. Ultrasound image measurements were used to determine that the skin stretches and changes in back muscle length were positively correlated, and demonstrate the feasibility of a proof of concept sensor. Besides, this back muscle training system can also estimate the prescribed strategy during Roman chair training. Furthermore, we explored the reproducibility of this sensor module during Roman chair training. In summary, this study developed a back muscle strengthening training system that is beneficial for establishing an effective training system.

## Figures and Tables

**Table 1.** Parameter settings for RF sputtering of the intermediate layer.

**Parameter**	**Value**
Target Material	Cr
Power	50 W
Sputter time	30 min
Process stress	1.8 × 10^−2^ torr
Environment temperature	27 °C

**Table 2.** Parameter settings for the RF sputtering of the electrode layer.

**Parameter**	**Value**
Target Material	Ag
Power	50 W
Sputter time	30 min
Process stress	1.8 × 10^−2^ torr
Environment temperature	27 °C

**Table 3.** Geometry of flexible sensors.

**Parameter**	**Value**
Length	40 ± 0.05 mm
Width	25 ± 0.03 mm
Substrate thickness	0.35 ± 0.002 mm
Sensor thickness	0.3516 ± 0.0002 mm

**Table 4.** Participant's physical data.

**Parameter**	**Value**
Height	164.5 cm
Weight	51.1 kg
Shoulder width	43.6 cm
Chest circumference	80.1 cm
Waist circumference	72.9 cm

**Table 5.** Results for ten repetitions of Roman chair back hyperextensions.

**Position**	**11**	**12**	**13**	**14**	**15**
Skin stretch (%)	0.5	1	3	1	1
Average resistance (Ω)	0.53 ± 0.74	1.21 ± 0.94	3.82 ± 1.47	1.33 ± 1.02	1.18 ± 0.74

**Position**	**6**	**7**	**8**	**9**	**10**

Skin stretch (%)	1	6.25	7	6	1
Average resistance (Ω)	1.42 ± 0.43	13.24 ± 2.02	15.95 ± 2.30	12.45 ± 0.69	1.47 ± 0.33

**Position**	**1**	**2**	**3**	**4**	**5**

Skin stretch (%)	25	32.5	32.5	32.5	27
Average resistance (Ω)	127.21 ± 12.26	156.37 ± 31.78	159.47 ± 29.15	158.64 ± 34.46	138.17 ± 22.42

**Table 6.** Comparison of changes in back muscle extension length.

	**Trapezius Back Skeletal Muscle Extension Length (cm)**	**Mid Erector Spinae Extension Length (cm)**	**Lower Erector Spinae Extension Length (cm)**
**Preparatory action**	2.47	2.94	2.94
**Anteflexion**	2.61	3.17	2.41
**Corresponding skin stretch**	0%–3%	3%–12%	20%–35%
